# Semaglutide and Resmetirom in Patients With Metabolic Dysfunction‐Associated Steatohepatitis and Fibrosis: An Exploratory Indirect Comparison

**DOI:** 10.1111/dom.71110

**Published:** 2026-07-15

**Authors:** Alessandro Mantovani, Giovanni Targher

**Affiliations:** ^1^ Department of Medicine University of Verona Verona Italy; ^2^ Metabolic Diseases Research Unit IRCCS Sacro Cuore ‐ Don Calabria Hospital Negrar di Valpolicella Italy

**Keywords:** effectiveness, fatty liver disease, GLP‐1 analogue, liver

## 1 Background

Metabolic dysfunction‐associated steatohepatitis (MASH) with moderate‐to‐advanced fibrosis (stages F2–F3) is a major public health challenge [1]. Recently, two medications have received conditional approval for treating MASH with moderate‐to‐advanced fibrosis. In March 2024, based on the phase 3 MAESTRO‐NASH trial [2], the US Food and Drug Administration (FDA) conditionally approved resmetirom, an oral liver‐directed thyroid hormone receptor‐β agonist. In August 2025, based on the phase 3 ESSENCE trial [3], the FDA approved subcutaneous semaglutide 2.4 mg/week, a glucagon‐like peptide‐1 receptor agonist.

Conditional approvals of resmetirom and semaglutide marked the entry of MASH into an era of available pharmacological options. In a recent commentary, Polyzos et al. [4] discussed the potential role of combining resmetirom and semaglutide (either concomitantly or sequentially) to treat adults with MASH and moderate‐to‐advanced fibrosis. However, in the absence of head‐to‐head comparative trials, only indirect treatment comparisons may provide preliminary evidence of comparative hepatic effectiveness. Therefore, we conducted an exploratory indirect comparison of histological liver endpoints between resmetirom and semaglutide in patients with MASH and fibrosis stages F2–F3 enrolled in the phase 3 placebo‐controlled MAESTRO‐NASH and ESSENCE trials, across the overall study population and predefined subgroups.

## 2 Methods

For this analysis, treatment effects for resmetirom and semaglutide were extracted as absolute differences versus placebo, using the estimated differences in proportions (EDP) that were reported in the phase 3 placebo‐controlled MAESTRO‐NASH trial for both resmetirom doses (80 mg and 100 mg/day) [2] and in the phase 3 placebo‐controlled ESSENCE trial for semaglutide (2.4 mg/week) [3], along with their corresponding 95% confidence intervals (CI). Data were collected for the overall population and prespecified subgroups, including sex (men vs. women), age (≥ 65 vs. < 65 years), body mass index (≥ 35 vs. < 35 kg/m^2^), type 2 diabetes status and baseline fibrosis stage (F2 or F3). Standard errors (SE) were calculated using the formula: SE = (upperCI − lowerCI)/3.92. Indirect comparisons between treatment effects were estimated as the risk difference (RD) between treatment effects of resmetirom and semaglutide, both in the overall population and in each subgroup, as follows: Delta RD = RD resmetirom − RD semaglutide. Assuming independence between trials, the variance of the indirect estimate was calculated as the sum of the variances of the two RDs. *Z*‐statistics, two‐sided *p* values and 95% CIs were subsequently derived for each subgroup comparison. All data are reported in Table S1.

## 3 Results

**FIGURE 1:**
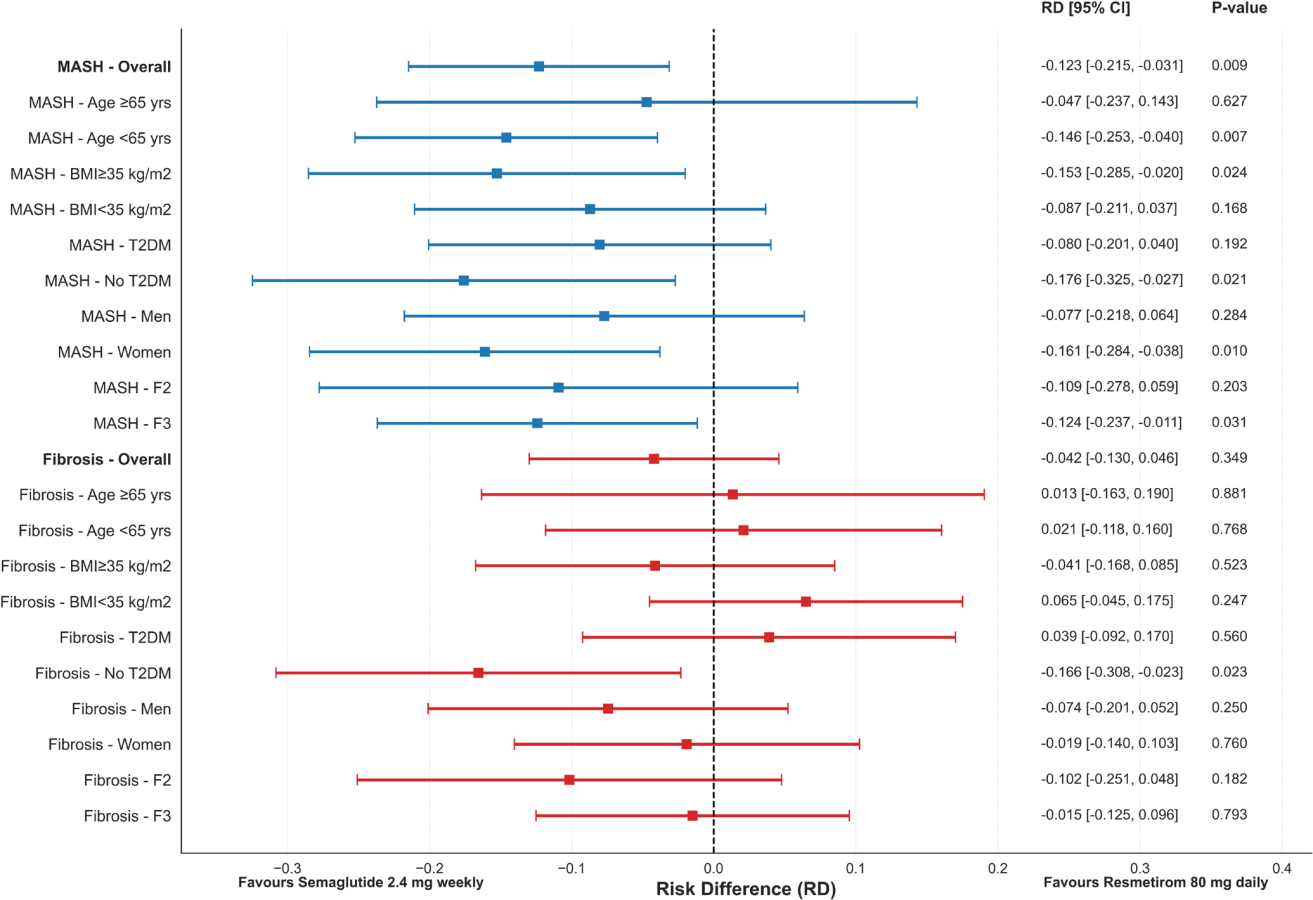
Indirect comparisons of treatment effects for histologic resolution of MASH without worsening of liver fibrosis (blue line) and for improvement in liver fibrosis without worsening of MASH (red line) between subcutaneous semaglutide 2.4 mg once weekly and resmetirom 80 mg once daily in the overall study population and in prespecified subgroups.

Figure 1 shows indirect comparisons of treatment effects between resmetirom 80 mg/day and semaglutide 2.4 mg/week for MASH resolution without worsening of fibrosis (blue line) and for liver fibrosis improvement without worsening of MASH (red line), in the overall population and prespecified subgroups. For MASH resolution, semaglutide showed a significantly greater benefit than resmetirom 80 mg/day in the overall population (RD: −0.123, 95% CI: −0.215 to −0.031) and in some subgroups, including patients aged < 65 years, women, patients with BMI ≥ 35 kg/m^2^, those without T2DM, and those with F3 fibrosis. No significant differences were observed between the two agents in the remaining subgroups. For fibrosis improvement, no significant differences were observed between treatments in the overall population or subgroups, except that semaglutide showed a greater benefit in patients without T2DM.

**FIGURE 2:**
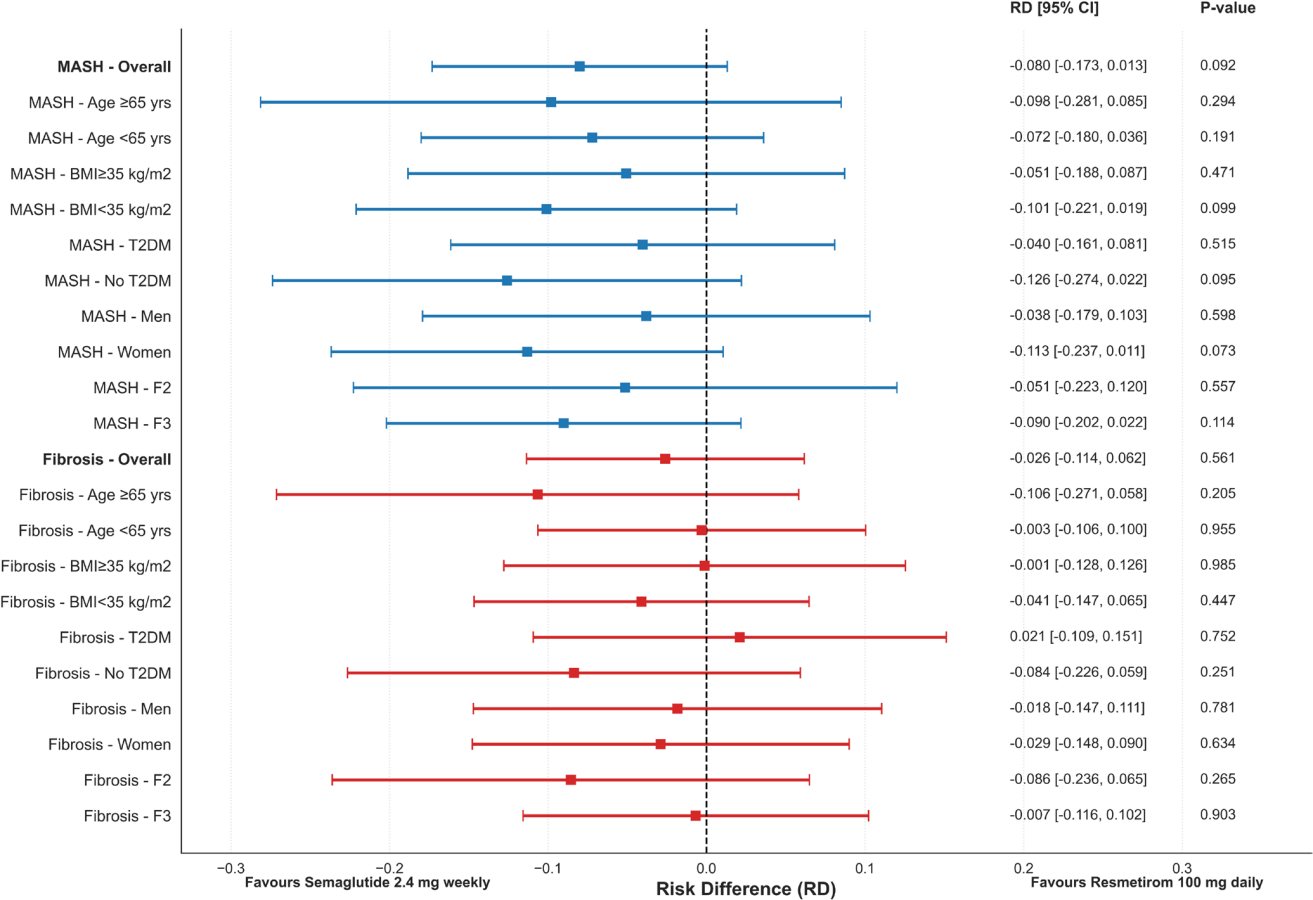
Indirect comparisons of treatment effects for histologic resolution of MASH without worsening of liver fibrosis (blue line) and for improvement in liver fibrosis without worsening of MASH (red line) between subcutaneous semaglutide 2.4 mg once weekly and resmetirom 100 mg once daily in the overall study population and in prespecified subgroups.

Figure 2 shows indirect comparisons of treatment effects between resmetirom 100 mg/day and semaglutide 2.4 mg/week for MASH resolution without worsening of fibrosis (blue line) and for liver fibrosis improvement without worsening of MASH (red line), in the overall population and in prespecified subgroups. For MASH resolution, semaglutide tended to confer greater benefit than resmetirom 100 mg/day in the overall population, although the difference was not significant (RD: −0.080; 95% CI: −0.173 to 0.013). Similar non‐significant trends favouring semaglutide were observed across predefined subgroups. For fibrosis improvement, no significant differences were observed between semaglutide and resmetirom 100 mg/day in the overall population or any subgroup.

## 4 Conclusions

These exploratory indirect comparisons offer preliminary insights into the comparative hepatic effectiveness of semaglutide and resmetirom for MASH and moderate‐to‐advanced fibrosis. Our findings suggest that semaglutide 2.4 mg/week confers greater benefit for MASH resolution without worsening of fibrosis than resmetirom, especially at a daily dose of 80 mg. Our results also suggest that compared with 80‐mg resmetirom, the greater benefit of semaglutide on MASH resolution is more pronounced in specific subgroups, including younger patients, women, patients with more severe obesity, those without T2DM, and those with F3 fibrosis. Although these findings should be interpreted with caution, they may generate hypotheses about the differential hepatic effectiveness of these two drugs across metabolic phenotypes and disease stages. Notably, semaglutide 2.4 mg/week and resmetirom (at both daily doses) appear broadly comparable with respect to improvement in liver fibrosis without worsening of MASH in both the overall population and prespecified subgroups.

Collectively, these findings support the notion that, despite distinct metabolic and molecular targets [5, 6], both agents ultimately converge on pathways relevant to the stabilization or regression of hepatic fibrosis. Because the hepatic fibrosis stage is the strongest histological predictor of liver‐related and overall mortality in individuals with MASH, the comparable anti‐fibrotic effects of semaglutide 2.4 mg/week and resmetirom (at both daily doses) may have important long‐term clinical implications. Moreover, these findings further support the hypothesis proposed by Polyzos et al. [4] that these MASH pharmacotherapies may be complementary, either as sequential or combination treatments.

Combination therapy is particularly attractive given the multifactorial nature of MASH pathogenesis [7]. MASH is characterized by a complex interplay among insulin resistance, adipose tissue dysfunction, lipotoxicity, oxidative stress, mitochondrial injury, inflammatory activation and fibrogenesis [1]. Therefore, a single pharmacological agent is unlikely to adequately target all relevant pathogenic pathways in every patient. In this context, semaglutide and resmetirom may theoretically provide additive or synergistic benefits through complementary mechanisms [4]. Future combination studies will therefore be of considerable interest.

Our findings should be interpreted with caution, given the limitations inherent in these analyses. First, this was an exploratory indirect comparison using aggregated published data rather than an individual patient‐level analysis. Consequently, adjustment for baseline differences between the MAESTRO‐NASH [2] and ESSENCE populations [3] was not possible. Differences in patient demographics, disease severity, metabolic characteristics, histological assessment and placebo response rates may have influenced the observed treatment effects. Second, indirect comparisons are inherently limited by the assumption of transitivity and comparability across trials. Although both trials enrolled obese individuals with MASH and fibrosis stages F2–F3, differences in study methodologies (including different imputation approaches for handling missing end‐of‐trial biopsy data) might affect interpretation. Similarly, differences in biopsy interpretation, central pathology reading or endpoint adjudication may introduce additional heterogeneity. Third, subgroup‐specific treatment effects were based on published aggregate estimates rather than on prospectively harmonized analyses. Although we compared the hepatic effectiveness of semaglutide and resmetirom in prespecified subgroups, confirmation in head‐to‐head randomized studies will be necessary before drawing definitive conclusions about subgroup‐specific hepatic efficacy.

In conclusion, this exploratory indirect comparison suggests that semaglutide 2.4 mg/week may confer slightly greater efficacy in achieving MASH resolution without worsening fibrosis than resmetirom (especially at 80 mg/day). Anti‐fibrotic hepatic effects appear broadly comparable between semaglutide 2.4 mg/week and resmetirom across both daily doses. We believe semaglutide should be prioritized to achieve sustained improvement in the systemic drivers of MASH, particularly in patients with obesity and/or type 2 diabetes. Conversely, resmetirom is best suited for patients with advanced fibrosis or an incomplete metabolic response, with combination strategies increasingly justified to target both systemic drivers and intrahepatic injury pathways. However, head‐to‐head comparative and combination trials are required to define optimal therapeutic strategies for MASH and moderate‐to‐advanced fibrosis.

## Funding

G.T. was supported in part by grants from the School of Medicine, University of Verona, Verona, Italy.

## Conflicts of Interest

The authors declare no conflicts of interest.

## Supporting information


**Table S1:** Indirect comparisons of treatment effects for histologic resolution of MASH without worsening of liver fibrosis and for improvement in liver fibrosis without worsening of MASH between resmetirom (80 and 100 mg/day orally) and subcutaneous semaglutide 2.4 mg once weekly in the overall study population and in prespecified subgroups.

## Data Availability

Data derived from public domain resources.
