# Supplementary material for: Semaglutide and Resmetirom in Patients With Metabolic Dysfunction‐Associated Steatohepatitis and Fibrosis: An Exploratory Indirect Comparison
Source: Diabetes Obes Metab. 2026 Jul 15;28(10):9707–10. doi: 10.1111/dom.71110 (PMC13538817; doi:10.1111/dom.71110)
Supplement: Supplementary file 1 [file DOM-28-9707-s001.docx]

**Supplementary Table 1**. Indirect comparisons of treatment effects for histologic resolution of MASH without worsening of liver fibrosis and for improvement in liver fibrosis without worsening of MASH between resmetirom (80 mg and 100 mg/day orally) and subcutaneous semaglutide 2.4 mg once weekly in the overall study population and in prespecified subgroups.

| **Subgroup(s)** | **EDP resmetirom** | **Lower 95%CI EDP resmetirom** | **Upper 95%CI EDP resmetirom** | **EDP semaglutide** | **Lower 95%CI EDP semaglutide** | **Upper 95%CI EDP semaglutide** | **Risk difference (RD)** | **Lower 95%CI RD** | **Upper 95%CI**  **RD** | **SE** | **Z** | **P-value** |
| --- | --- | --- | --- | --- | --- | --- | --- | --- | --- | --- | --- | --- |
| **Resmetirom 80 mg/day vs. Semaglutide 2.4 mg/week** | | | | | | | | | | | | |
| **Resolution of MASH without worsening of fibrosis** | | | | | | | | | | | | |
| Overall population | 0.164 | 0.11 | 0.22 | 0.29 | 0.21 | 0.36 | -0.123 | -0.2147 | -0.0313 | 0.0468 | -2.63 | 0.0085 |
| Age ≥65 yrs | 0.262 | 0.15 | 0.38 | 0.31 | 0.16 | 0.46 | -0.0471 | -0.2373 | 0.1431 | 0.097 | -0.485 | 0.6274 |
| Age <65 yrs | 0.134 | 0.07 | 0.2 | 0.28 | 0.19 | 0.37 | -0.146 | -0.2525 | -0.0395 | 0.0544 | -2.686 | 0.0072 |
| BMI≥35 kg/m^2^ | 0.098 | 0.03 | 0.17 | 0.25 | 0.14 | 0.36 | -0.1526 | -0.2852 | -0.02 | 0.0677 | -2.255 | 0.0241 |
| BMI<35 kg/m^2^ | 0.224 | 0.14 | 0.31 | 0.31 | 0.22 | 0.4 | -0.087 | -0.2106 | 0.0366 | 0.0631 | -1.379 | 0.1678 |
| T2DM | 0.164 | 0.1 | 0.23 | 0.24 | 0.14 | 0.35 | -0.0802 | -0.2007 | 0.0403 | 0.0615 | -1.304 | 0.1921 |
| No T2DM | 0.164 | 0.07 | 0.26 | 0.34 | 0.23 | 0.45 | -0.1758 | -0.3246 | -0.027 | 0.0759 | -2.315 | 0.0206 |
| Men | 0.181 | 0.1 | 0.27 | 0.26 | 0.14 | 0.37 | -0.077 | -0.2178 | 0.0638 | 0.0718 | -1.072 | 0.2837 |
| Women | 0.153 | 0.08 | 0.23 | 0.31 | 0.21 | 0.41 | -0.1611 | -0.2844 | -0.0378 | 0.0629 | -2.562 | 0.0104 |
| F2 stage | 0.176 | 0.08 | 0.27 | 0.29 | 0.15 | 0.42 | -0.1093 | -0.2777 | 0.0591 | 0.0859 | -1.272 | 0.2034 |
| F3 stage | 0.162 | 0.09 | 0.23 | 0.29 | 0.2 | 0.38 | -0.1241 | -0.2368 | -0.0114 | 0.0575 | -2.159 | 0.0308 |
| **Improvement in liver fibrosis without worsening of MASH** | | | | | | | | | | | | |
| Overall population | 0.102 | 0.05 | 0.16 | 0.14 | 0.07 | 0.21 | -0.042 | -0.1299 | 0.0459 | 0.0449 | -0.936 | 0.3492 |
| Age ≥65 yrs | 0.166 | 0.05 | 0.28 | 0.15 | 0.02 | 0.29 | 0.0135 | -0.1634 | 0.1904 | 0.0902 | 0.15 | 0.8811 |
| Age <65 yrs | 0.166 | 0.05 | 0.28 | 0.14 | 0.07 | 0.23 | 0.021 | -0.1183 | 0.1603 | 0.0711 | 0.296 | 0.7676 |
| BMI ≥35 kg/m^2^ | 0.07 | -0.01 | 0.15 | 0.11 | 0.01 | 0.21 | -0.0412 | -0.1676 | 0.0852 | 0.0645 | -0.639 | 0.5228 |
| BMI <35 kg/m^2^ | 0.224 | 0.14 | 0.31 | 0.16 | 0.09 | 0.23 | 0.065 | -0.0451 | 0.1751 | 0.0562 | 1.157 | 0.2473 |
| T2DM | 0.128 | 0.06 | 0.19 | 0.09 | -0.05 | 0.18 | 0.039 | -0.0922 | 0.1702 | 0.067 | 0.582 | 0.5603 |
| No T2DM | 0.05 | -0.05 | 0.15 | 0.22 | 0.12 | 0.32 | -0.1656 | -0.3081 | -0.0231 | 0.0727 | -2.279 | 0.0227 |
| Men | 0.095 | 0.02 | 0.17 | 0.17 | 0.07 | 0.27 | -0.0743 | -0.201 | 0.0524 | 0.0646 | -1.15 | 0.2503 |
| Women | 0.106 | 0.03 | 0.18 | 0.12 | 0.03 | 0.22 | -0.0189 | -0.1404 | 0.1026 | 0.062 | -0.305 | 0.7604 |
| F2 stage | 0.098 | 0.01 | 0.19 | 0.2 | 0.08 | 0.32 | -0.1016 | -0.2509 | 0.0477 | 0.0762 | -1.334 | 0.1822 |
| F3 stage | 0.105 | 0.03 | 0.18 | 0.12 | 0.04 | 0.2 | -0.0148 | -0.1251 | 0.0955 | 0.0563 | -0.263 | 0.7926 |
| **Resmetirom 100 mg/day vs. Semaglutide 2.4 mg/week** | | | | | | | | | | | | |
| **Resolution of MASH without worsening of fibrosis** | | | | | | | | | | | | |
| Overall population | 0.207 | 0.15 | 0.26 | 0.29 | 0.21 | 0.36 | -0.08 | -0.1731 | 0.0131 | 0.0475 | -1.684 | 0.0922 |
| Age ≥65 yrs | 0.211 | 0.11 | 0.32 | 0.31 | 0.16 | 0.46 | -0.0981 | -0.2812 | 0.085 | 0.0934 | -1.05 | 0.2936 |
| Age <65 yrs | 0.208 | 0.14 | 0.27 | 0.28 | 0.19 | 0.37 | -0.072 | -0.18 | 0.036 | 0.0551 | -1.307 | 0.1913 |
| BMI ≥35 kg/m^2^ | 0.2 | 0.12 | 0.28 | 0.25 | 0.14 | 0.36 | -0.0506 | -0.1883 | 0.0871 | 0.0703 | -0.72 | 0.4714 |
| BMI <35 kg/m^2^ | 0.21 | 0.13 | 0.29 | 0.31 | 0.22 | 0.4 | -0.101 | -0.221 | 0.019 | 0.0612 | -1.649 | 0.0991 |
| T2DM | 0.204 | 0.14 | 0.27 | 0.24 | 0.14 | 0.35 | -0.0402 | -0.1613 | 0.0809 | 0.0618 | -0.651 | 0.5151 |
| No T2DM | 0.214 | 0.12 | 0.31 | 0.34 | 0.23 | 0.45 | -0.1258 | -0.2737 | 0.0221 | 0.0754 | -1.668 | 0.0954 |
| Men | 0.22 | 0.14 | 0.3 | 0.26 | 0.14 | 0.37 | -0.038 | -0.1791 | 0.1031 | 0.072 | -0.528 | 0.5976 |
| Women | 0.201 | 0.13 | 0.27 | 0.31 | 0.21 | 0.41 | -0.1131 | -0.2367 | 0.0105 | 0.063 | -1.794 | 0.0728 |
| F2 stage | 0.234 | 0.13 | 0.34 | 0.29 | 0.15 | 0.42 | -0.0513 | -0.2227 | 0.1201 | 0.0874 | -0.587 | 0.5575 |
| F3 stage | 0.196 | 0.13 | 0.26 | 0.29 | 0.2 | 0.38 | -0.0901 | -0.2019 | 0.0217 | 0.057 | -1.58 | 0.1141 |
| **Improvement in liver fibrosis without worsening of MASH** | | | | | | | | | | | | |
| Overall population | 0.118 | 0.06 | 0.17 | 0.14 | 0.07 | 0.21 | -0.026 | -0.1136 | 0.0616 | 0.0447 | -0.582 | 0.5608 |
| Age ≥65 yrs | 0.046 | -0.05 | 0.14 | 0.15 | 0.02 | 0.29 | -0.1065 | -0.2712 | 0.0582 | 0.084 | -1.267 | 0.205 |
| Age <65 yrs | 0.142 | 0.08 | 0.21 | 0.14 | 0.07 | 0.23 | -0.003 | -0.1064 | 0.1004 | 0.0528 | -0.057 | 0.9546 |
| BMI ≥35 kg/m^2^ | 0.11 | 0.03 | 0.19 | 0.11 | 0.01 | 0.21 | -0.0012 | -0.1279 | 0.1255 | 0.0646 | -0.019 | 0.9852 |
| BMI <35 kg/m^2^ | 0.118 | 0.04 | 0.19 | 0.16 | 0.09 | 0.23 | -0.041 | -0.1467 | 0.0647 | 0.0539 | -0.76 | 0.4472 |
| T2DM | 0.11 | 0.05 | 0.17 | 0.09 | -0.05 | 0.18 | 0.021 | -0.1093 | 0.1513 | 0.0665 | 0.316 | 0.752 |
| No T2DM | 0.132 | 0.03 | 0.23 | 0.22 | 0.12 | 0.32 | -0.0836 | -0.2264 | 0.0592 | 0.0729 | -1.147 | 0.2512 |
| Men | 0.151 | 0.07 | 0.23 | 0.17 | 0.07 | 0.27 | -0.0183 | -0.1471 | 0.1105 | 0.0657 | -0.278 | 0.7807 |
| Women | 0.096 | 0.02 | 0.17 | 0.12 | 0.03 | 0.22 | -0.0289 | -0.1478 | 0.09 | 0.0607 | -0.476 | 0.6339 |
| F2 stage | 0.114 | 0.02 | 0.21 | 0.2 | 0.08 | 0.32 | -0.0856 | -0.2361 | 0.0649 | 0.0768 | -1.115 | 0.265 |
| F3 stage | 0.113 | 0.04 | 0.18 | 0.12 | 0.04 | 0.2 | -0.0068 | -0.1158 | 0.1022 | 0.0556 | -0.122 | 0.9027 |

*Abbreviations*: BMI, body mass index; CI, confidence interval; EDP, estimated differences in proportions; MASH, metabolic dysfunction-associated steatohepatitis; SE, standard error; T2DM, type 2 diabetes mellitus.
Note: Because some subgroups of interest (i.e., age <65 years and BMI <35 kg/m²) were not directly reported in the supplementary materials of the ESSENCE trial, whereas they were available in the MAESTRO-NASH trial, we indirectly estimated the EDP for the ESSENCE trial by combining data from the reported subgroup categories (i.e., age <45 years and 45–65 years; BMI <27 kg/m², 27–30 kg/m², and 30–35 kg/m²) using the information that were provided in Figures S5 and S6 of the article.
